# Prevalence of high-risk human papillomavirus infection and cervical lesions among female migrant head porters (*kayayei*) in Accra, Ghana: a pilot cross-sectional study

**DOI:** 10.1186/s12889-024-18094-9

**Published:** 2024-03-01

**Authors:** Ethel Tekpor, Kofi Effah, Jerry Sifa, Joseph Emmanuel Amuah, Nana Owusu Mensah Essel, Seyram Kemawor, Comfort Mawusi Wormenor, Edna Sesenu, Stephen Danyo, Patrick Kafui Akakpo

**Affiliations:** 1 Cervical Cancer Prevention and Training Centre, Catholic Hospital, Battor, Ghana; 2 University Health Services, University of Ghana, Accra, Ghana; 3 School of Epidemiology and Public Health, Faculty of Medicine, University of Ottawa, Ottawa, ON Canada; 4 Department of Emergency Medicine, College of Health Sciences, Faculty of Medicine and Dentistry, University of Alberta, 730 University Terrace, T6G 2T4 Edmonton, AB Canada; 5 Department of Pathology, School of Medical Sciences, University of Cape Coast, Cape Coast, Ghana

**Keywords:** Human papillomavirus infections, Cervical cancer, Cancer screening, Internal migrant workers, Women’s health, Health equality

## Abstract

**Background:**

Little attention has been given to the risk of high-risk human papillomavirus (hr-HPV) infection and cervical precancerous lesions among female migrant head porters (*kayayei*) in Ghana, as a vulnerable group, and to promote cervical screening in these women. This pilot study aimed to determine the prevalence of hr-HPV infection and cervical lesions among *kayayei* in Accra, the capital of the Greater Accra Region of Ghana and to describe our approach to triaging and treating these women.

**Methods:**

This descriptive cross-sectional cohort study involved the screening of 63 *kayayei* aged ≥ 18 years at the Tema Station and Agbogbloshie markets in March 2022 and May 2022. Concurrent hr-HPV DNA testing (with the MA-6000 platform) and visual inspection with acetic acid (VIA) was performed. We present prevalence estimates for hr-HPV DNA positivity and VIA ‘positivity’ as rates, together with their 95% confidence intervals (CIs). We performed univariate and multivariable nominal logistic regression to explore factors associated with hr-HPV infection.

**Results:**

Gross vulvovaginal inspection revealed vulval warts in 3 (5.0%) and vaginal warts in 2 (3.3%) women. Overall, the rate of hr-HPV positivity was 33.3% (95% CI, 21.7–46.7), whereas the VIA ‘positivity’ rate was 8.3% (95% CI, 2.8–18.4). In the univariate logistic regression analysis, none of the sociodemographic and clinical variables assessed, including age, number of prior pregnancies, parity, past contraceptive use, or the presence of abnormal vaginal discharge showed statistically significant association with hr-HPV positivity. After controlling for age and past contraceptive use, only having fewer than two prior pregnancies (compared to having ≥ 2) was independently associated with reduced odds of hr-HPV infection (adjusted odds ratio, 0.11; 95% CI, 0.02–0.69).

**Conclusion:**

In this relatively young cohort with a high hr-HPV positivity rate of 33.3% and 8.3% of women showing cervical lesions on visual inspection, we posit that *kayayei* may have an increased risk of developing cervical cancer if their accessibility to cervical precancer screening services is not increased.

## Background

Globally, cervical cancer ranks fourth for cancer incidence and mortality among women [1], estimated to occur in one in 70 women before the age of 75 years [2]. Comparatively, in Ghana, cervical cancer has an estimated cumulative risk of 3% among women aged < 75 years [3], and ranks second for incidence after breast cancer and first for cancer-associated deaths [4]. Almost all cervical cancers result from persistent high-risk human papillomavirus (hr-HPV) infection, and the disease is largely preventable through regular screening due to its long latency period [5]. Migrant women are known to be more vulnerable to cervical cancer [6]. A number of studies emanating from high-income settings have pointed to a higher incidence of cervical cancer among migrants compared to native women [7–11]. In addition, migrant women have recorded lower participation rates in cervical cancer screening programs, increasing their risk of presenting with more advanced lesions and poorer treatment outcomes [12, 13].

Specific to the Ghanaian context, high levels of poverty in the Northern parts often compel people (in particular, young women) to migrate southward to major cities to gain employment with the intent of freeing themselves from economic destitution [14, 15]. Upon arriving, these girls often work as head porters (locally known as ‘*kayayei*’; singular, ‘*kayayo*’, coined from ‘*kaya*’ meaning ‘load’ in Hausa, and ‘*yoo*’, meaning ‘girl’ in the Ga language), making money by carrying the heavy loads of people who purchase items in marketplaces [16, 17]. Research has shown that these young women (often teenagers) take up these roles as the easiest way of pursuing their economic dreams and maintaining their livelihood [18]. Some *kayayei* also do not migrate willingly, but are coerced by their families, to remit relatives back home [19, 20]. In providing cheap labor to local patrons, many *kayayei* live hand-to-mouth as they do not make enough to save and move back home [21], and are thus faced with a number of social and health challenges. Due to their poor economic circumstances, *kayayei* tend to be unable to secure shelter, and so sleep on the streets, at market squares, and at bus stations [22], resulting in accompanying issues of safety (such as robbery, further compounding their economic situation) and rape (leading to sexually transmitted diseases and unwanted pregnancies) [23]. There have also been reports of *kayayei* experiencing physical assault and injuries from patrons and market traders alike, kidnapping, and engaging in drug and alcohol addiction while they strive to survive on the streets [16, 24]. The National *Kayayei* Dialogue held in 2021 with support from the United Nations Population Fund revealed that there are more than 100,000 *kayayei* in Ghana, with more than 70% working in the capital, Accra [25, 26].

In addition to the aforementioned attribution of the *kayayo* phenomenon to economic reasons, others believe that it is caused by a need to escape forced marriages and female genital mutilation, the perceived glamour of urban life, as well as a need to amass property for marriage [27]. Regardless of the motivation for engaging in the *kayayo* business, the health risks that accompany this phenomenon are undeniable, compounded by the previously described vulnerability to sexual violence and sexually transmitted diseases. To worsen their predicament, *kayayei* tend to have poor access to health services and a low rate of contraceptive use [27, 28] and have been reported to be particularly susceptible to transition into prostitution (at a rate as high as 56%) when their economic hopes for migrating to the south are dashed [29].

In describing the health status of these young female migrants, accurate data are usually fragmented or difficult to apply to policymaking. Recent research pertaining to the reproductive health needs of these vulnerable *kayayei* in Ghana has primarily focused on barriers to contraceptive use, health insurance coverage, care-seeking behavior, and risks of infectious diseases [27, 28, 30–32]. Little attention has been given to the risks of hr-HPV, specifically, and cervical precancerous lesions among *kayayei*, as a vulnerable group, and to investigate cervical screening among them. While the World Health Organization (WHO) recommends HPV-based cervical precancer screening for low-resource countries like Ghana, the country has no tailored cervical screening policy based on these recommendations. Developing such a guideline requires up-to-date evidence and estimates from specific vulnerable and marginalized populations, such as female prisoners (which our group recently investigated [33]) and internal female migrants (*kayayei*), apart from prevalence estimates from females in the general population. In the end, such research is crucial to informing intervention development that targets cervical precancer screening uptake and completion in this group, thus reducing disparities in cervical cancer incidence. Therefore, we conducted this pilot study to determine the prevalence of hr-HPV infection and cervical lesions among female migrant head porters (*kayayei*) working in the Greater Accra Region of Ghana and to describe our approach to triaging and treating these women.

## Methods

### Study design and participants

We conducted this retrospective descriptive cross-sectional cohort study to determine the prevalence of hr-HPV infection and cervical lesions among *kayayei* in the Greater Accra Region of Ghana. This study involved the screening of 63 head porters aged ≥ 18 years recruited at the Tema Station (*n* = 13) and Agbogbloshie market (*n* = 50) in the Central Business District of the Accra Metropolis in Ghana on 20th March 2022 and 8th May 2022. The women were screened under the Ghanaian arm of the mPharma 10,000 Women Initiative that provided free cervical precancer screening with HPV DNA testing to 10,000 women in Ghana and Nigeria. The screening sessions were held on two Sundays since a majority of the *kayayei* worked during weekdays and Saturdays. *Kayayei* who were willing and able to participate in the screening sessions were included in the study.

### Eligibility criteria

The inclusion criteria were as follows: any female migrant head porter older than 25 years; intact uterus; verbal consent to undergo cervical precancer screening with HPV DNA testing and visual inspection with acetic acid (VIA); and willingness to undergo single-visit management if necessary. The exclusion criteria were inability or unwillingness to consent to screening procedures, retracted cervix, and prior gynecological procedures that would make it impossible to collect cervical tissue (such as total abdominal hysterectomy). Younger women (18 to 24 years old) who were parous, or had been or were sexually active and opted for screening after our education on risk factors for cervical cancer were also offered screening, and were included in the study.

### Study settings

Old Fadama Market (popularly known as Agbogbloshie Market) is situated in a densely populated resource-poor area. A large majority of the residents of Agbogbloshie have no access to potable water and sanitation [34]. Residents live in a blend of concrete buildings and wooden structures. In many cases, people living within a single unit are unrelated. The area is situated close to the banks of the Korle river and is prone to floods, which have been responsible for significant morbidity and mortality [34]. Tema Station is a major lorry terminal in the heart of the capital, and acts as a central point within and outside the boundaries of Accra, Ghana. Due to its proximity to the Central Business District, which is a high-interest zone for many socioeconomic activities, Tema Station tends to accommodate a considerably high number of market patrons and commuters [35].

### Ethical considerations

The study complied with the Declaration of Helsinki (1964) and its later amendments. All study participants provided verbal informed consent before questionnaire administration, cervical sample collection, and visual inspection procedures. The consent procedure was approved by the Ethical Review Committee of the Catholic Hospital, Battor (approval no. CHB-ERC 0120/06/22), which also gave the researchers permission to publish the study findings retrospectively.

### Sample size

No sample size calculation was performed because the work was not initially conducted in the context of a research study and was piloted to explore outcomes of cervical screening among the *kayayei*. Instead, we included a convenience sample of all *kayayei* who were eligible and consented to participate in the screening exercises. Moreover, there was a scarcity of research focusing on the risk of cervical precancer and cancer among *kayayei*, which would be essential for providing an objective basis for such a calculation.

### Data collection and storage

All sociodemographic data presented were collected routinely as part of the screening process after obtaining verbal informed consent using a structured questionnaire administered by trained nurses who performed the screening. Following screening, questionnaire data and screening outcomes were entered into REDCap version 11.0.3 (Vanderbilt University, Nashville, TN, USA), and stored securely in databases managed by the Cervical Cancer Prevention and Training Centre (CCPTC), Battor, Ghana. All personal data were anonymized prior to the analyses.

### Study variables and outcomes

We extracted participant data from the databases, including sociodemographic variables such as age, marital status, number of children, highest level of education, monthly income, National Health Insurance Scheme (NHIS) coverage, and religious faith. Data regarding self-reported risk factors were also extracted, including HIV status, current contraceptive use, and smoking status (ever/current). The outcomes of interest were a positive hr-HPV DNA test determined using the MA-6000 platform (Sansure Biotech Inc., Hunan, China) or the presence of clinically relevant lesions and/or major/minor changes on VIA.

### Cervical screening (VIA) procedure and HPV sample collection

All participants were subjected to concurrent hr-HPV DNA testing with the MA-6000 platform and VIA. Cervical visual inspection and sample collection were performed by trained nurses after placing the women in the dorsal lithotomy position. A sterile vaginal speculum was inserted to expose the cervix and a cytobrush or cotton-tipped applicator was used to take cervical samples, which was then placed in a sample collection tube and submitted to the laboratory for testing.

In the same session, VIA was performed by the trained nurses. During the procedure, the cervix was inspected carefully for abnormal changes under a good light source after applying 5% acetic acid and waiting for 90–120 s. The results of VIA were described qualitatively as ‘negative’ or ‘positive’ (presence of aceto-whitening at the transformation zone). Colposcopy with the Enhanced Visual Assessment (EVA) system (MobileODT, Tel Aviv, Israel) was immediately performed for *kayayei* who showed significant changes/lesions on VIA to obtain images for quality assurance. All *kayayei* were triaged and managed as per our algorithms for cervical screening with VIA/mobile colposcopy which have been described elsewhere [33].

### Laboratory testing: MA-6000 DNA extraction and PCR assay

Cervicovaginal specimens were processed for MA-6000 testing in strict accordance with the manufacturer’s instructions [36], and as described in a prior publication [37]. Briefly, a pure fraction of DNA was isolated in solution, to which a sample release reagent was added, mixed, and allowed to incubate at 25 °C for 10 min. Based on the manufacturer’s amplification protocols, PCR reactions (50 µL each) were set up and run on the MA-6000 device for a total of 45 cycles: at 50 °C for 2 min, 94 °C for 5 min, 94 °C for 5 s, and 57 °C for 30 s. During the amplification step at 57 °C for 30 s fluorescence data were collected. The semi-quantitative MA-6000 kit is configured to detect four dyes: FAM (HPV 18); HEX (to detect β-globin as the internal control); CY5 (HPV 16); and ROX (which determines HPV 31/33/35/39/45/51/52/53/56/58/59/66/68, without distinction, as hr-HPV). The test results were read in conformity with the manufacturer’s guidelines.

### Statistical methods

We present descriptive statistics for categorical variables as frequencies and percentages. Continuous variables are described as medians with their ranges. The distributions of age and number of prior pregnancies among the *kayayei*, stratified by hr-HPV test result were compared using the Mann–Whitney *U* test. Prevalence estimates for hr-HPV DNA positivity and VIA ‘positivity’ are presented in rate form, together with their binomial 95% confidence intervals (CIs). We further explored the association between hr-HPV positivity and selected sociodemographic and clinical variables using univariate and multivariable binary logistic regression. The multivariable analysis was performed using the backward elimination procedure with an arbitrary threshold of *p*-value = 0.25. Effect estimates from the exploratory regression analyses are reported as crude odds ratios (ORs) and adjusted ORs (aORs) with 95% CIs. Due to the low number of head porters with positive findings on visual inspection, we did not explore factors associated with VIA or EVA positivity. All statistical analyses were performed using Stata version 15 (StataCorp LLC, College Station, TX, USA). The null hypothesis was rejected at a two-tailed alpha level of 5%.

## Results

### Participant recruitment and selection

**Fig. 1:**
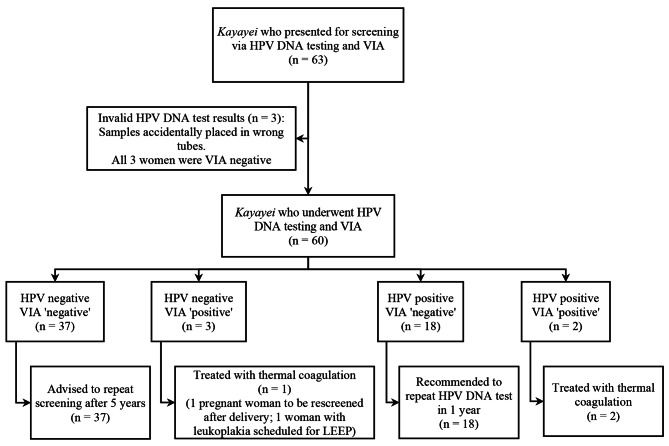
Flow chart for cervical precancer screening among the migrant head porters. HPV, human papillomavirus; VIA, visual inspection with acetic acid; LEEP, loop electrosurgical excision procedure

In total, 63 migrant head porters presented for cervical precancer screening during the two days, the characteristics of whom are shown in Table 1. A majority of them (79%) were screened at Agbogbloshie market, while the remainder (21%) were screened at Tema Station. Three of the initial 63 *kayayei* had invalid HPV test results because their cervical samples were mistakenly placed in the wrong tubes; all of these three women showed negative VIA findings. Thus, a total of 60 women were screened concurrently with VIA and HPV DNA testing. All 63 migrant head porters were included in the descriptive analysis, whereas 60 out of the 63 were included in the risk factor analysis after excluding the 3 women with invalid hr-HPV DNA test results (Fig. 1).

### Sociodemographic and clinical characteristics of the migrant head porters

The women had a median age of 23 years and were mostly married (59%) or had a steady partner (27%). A large majority of the participants had at least one child (81%) and had completed at least elementary school (71%). 91% of the head porters belonged to the Islamic faith, followed by Christianity (8%), and the African traditional religion (2%). As self-reported risk factors, most of the head porters had unknown HIV statuses or had never tested for HIV (87%), did not use any form of contraception (86%), and had never smoked (100%). Close to 80% of the *kayayei* covered their medical bills themselves or through the NHIS, while 3% and 6% relied on relatives and other sources, respectively (Table 1). In terms of gynecologic history, 5% had experienced postcoital bleeding, 18% had experienced abnormal vaginal discharge, and only one (2%) had undergone prior gynecological surgery. None (0%) of the participants had received prior cervical screening or prior HPV vaccination.

### Screening characteristics and outcomes of the migrant head porters

**Fig. 2:**
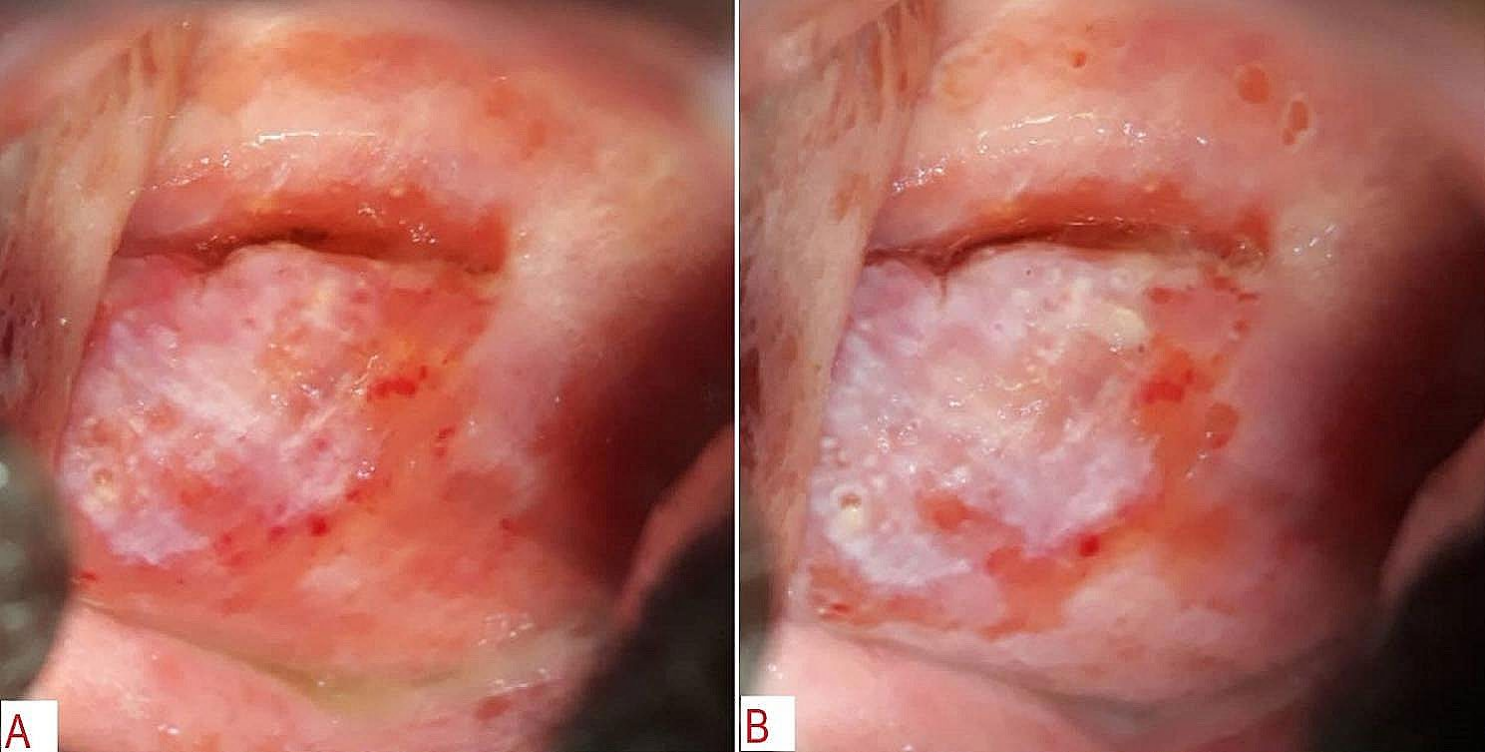
Colposcopic images of a 30-year-old migrant head porter, para 3: **(A)** before applying acetic acid and **(B)** after applying acetic acid. HPV DNA testing was performed alongside visual inspection with acetic acid followed by mobile colposcopy with the Enhanced Visual Assessment (EVA) system in the same setting. Colposcopy was performed because there was leukoplakia (white patch) on the cervix even before acetic acid was applied. HPV DNA test result–negative for hr-HPV; colposcopy findings–transformation zone type 3, leukoplakia with aceto-whitening on the posterior lip of the cervix. Plan: Scheduled for LEEP

A flow chart of the screening and HPV test results of the *kayayei* is presented in Fig. 1, alongside the treatments offered to those with clinically significant lesions. Most of the head porters showed normal findings on gross vulval (95%) or vaginal (97%) inspection. Vulvovaginal inspection revealed vulval warts in 3 (5.0%) and vaginal warts in 2 (3.3%), while VIA yielded positive findings in 5 (8.3%). The commonest transformation zone types on visual inspection were types 2 (41%) and 3 (46%). Overall, the rate of hr-HPV positivity was 33.3% (95% CI, 21.7–46.7), whereas the VIA ‘positivity’ rate was 8.3% (95% CI, 2.8–18.4) (Table 2). As outcomes of concurrent testing, a majority (*n* = 37, 61.7%) were concurrently negative, whereas 2 (3.3%) women tested concurrently positive. The cervical lesions of both women who tested concurrently VIA and HPV positive were treated by thermal coagulation. Three (5%) head porters tested hr-HPV negative but VIA positive. One was treated via thermal coagulation, another with leukoplakia was scheduled for a LEEP (Fig. 2), while yet another (a pregnant woman) was scheduled to undergo rescreening after delivery.

**Table 1:** Sociodemographic and clinical characteristics of 63 migrant head porters who underwent cervical screening via concurrent hr-HPV DNA testing and VIA

Sociodemographic variables	Estimate
Age, years; median (range)	23.0 (19–40)
No. of prior pregnancies, median (range)	1 (0–5)
No. of children, median (range)	0 (0–2)
Religion, n (%)	
Christianity	5 (7.9)
Islamic	57 (90.5)
African traditional religion	1 (1.6)
Marital status, n (%)	
Single	7 (11.1)
Has a steady partner	17 (27.0)
Married	37 (58.7)
Divorced	1 (1.6)
Widowed	1 (1.6)
Highest level of education, n (%)	
No formal education	18 (28.6)
Elementary education	20 (31.8)
Secondary education	25 (41.7)
Monthly income, GH¢; n (%)	
<100	30 (47.6)
100–250	23 (36.5)
251–500	4 (6.4)
>500	5 (7.9)
Unable to tell	1 (1.6)
Source of funds for medical bill payment^¥^	
Self, n (%)	50 (79.4)
Relatives, n (%)	2 (3.2)
Current/former employer, n (%)	0 (0.0)
NHIS, n (%)	50 (79.4)
Other, n (%)	4 (6.4)
Ever/current smoking, n (%)	0 (0.0)
Ever/current alcohol consumption, n (%)	0 (0.0)
Past contraceptive use^¥^, n (%)	27 (42.9)
Condoms, n (%)	2 (3.2)
Combined pill, n (%)	2 (3.2)
Depot-Provera, n (%)	16 (25.4)
Implant, n (%)	7 (11.1)
Current contraceptive use, n (%)	9 (14.3)
**Clinical characteristics**	
HIV status, n (%)	
Negative	8 (12.7)
Unknown/never tested	55 (87.3)
Gynecological history^¥^	
Postcoital bleeding, n (%)	3 (4.8)
Abnormal vaginal discharge, n (%)	11 (17.5)
Intermenstrual bleeding, n (%)	0 (0.0)
Known medical condition, n (%)	5 (7.9)
Prior gynecological surgery, n (%)	1 (1.6)
Previous cervical screening, n (%)	0 (0.0)
Prior HPV vaccination, n (%)	0 (0.0)

HIV, human immunodeficiency virus

^¥^ Multiple-choice item

**Table 2:** Screening characteristics and outcomes of 63 migrant head porters who underwent cervical screening via concurrent hr-HPV DNA testing and VIA

Screening characteristic	Estimate
Normal vulval inspection findings, n (%)	60 (95.2)
Normal vaginal inspection findings, n (%)	61 (96.8)
Cervical TZ type on visual inspection (VIA) ^α^, n (%)	
1	8 (12.7)
2	26 (41.3)
3	29 (46.0)
**Screening outcome (prevalence estimates)**	
Overall hr-HPV positive^¥^, % (95% CI)	33.3 (21.7–46.7)
VIA ‘positive’, % (95% CI)	8.3 (2.8–18.4)

hr-HPV, high-risk human papillomavirus; TZ, transformation zone; VIA, visual inspection with acetic acid; CI, confidence interval

^¥^ Denominator: 60 migrant head porters with valid hr-HPV DNA test results

^α^ Transformation zone types

TZ1: The entire circumference of the squamocolumnar junction is visible; fully ectocervical.

TZ2: The entire circumference of the squamocolumnar junction is visible; partly or fully endocervical.

TZ3: The entire circumference of the squamocolumnar junction is not visible; partly or fully endocervical.

### Exploratory analysis of factors associated with hr-HPV infection among the *kayayei*

Among the female migrant head porters, there were no significant differences between those with and without hr-HPV infection regarding age and number of prior pregnancies. In the univariate logistic regression analysis, none of the sociodemographic and clinical variables assessed, including age (OR, 0.98; 95% CI, 0.87–1.10), number of prior pregnancies (OR, 1.18; 95% CI, 0.80–1.74), parity (OR, 1.05; 95% CI, 0.36–3.04), past contraceptive use (OR, 1.76; 95% CI, 0.60–5.23), and the presence of abnormal vaginal discharge (OR, 1.41; 95% CI, 0.36–5.53) showed significant associations with hr-HPV positivity. In the adjusted logistic regression analysis (Table 3), after controlling for age and past contraceptive use, only having fewer than two prior pregnancies (compared to having ≥ 2) was independently associated with reduced odds of hr-HPV infection among the migrant head porters (aOR, 0.11; 95% CI, 0.02–0.69). While unit increases in age were also associated with reduced odds of hr-HPV infection in the adjusted analysis, this association was only marginally significant (aOR, 0.80; 95% CI, 0.65–1.00) (Table 3).

### Triage, treatment, and follow-up strategies

Due to resource limitations, inadequate funding, and to mitigate the risks associated with loss to follow-up, a ‘screen and treat’ approach was used as much as possible in screening, with VIA and mobile colposcopy as the primary screening test in a single visit, followed by treatment. Although hr-HPV testing was performed, since the results were not available in real time, it was used to confirm the decision to treat in retrospect and help make follow-up recommendations. Women with lesions on the cervix (minor/major changes) were given the option of treatment on site even before the HPV results came in. For women who had major change (the HPV results were not available on the field), the standard approach would be to perform a biopsy which would then guide management. An alternative approach, if they could afford it, would have been to perform a ‘diagnostic loop electrosurgical excision procedure (LEEP)’ which has the added benefit of being both diagnostic and therapeutic and reduces the cost that comes with multiple visits and double payment for histopathology. Since the *kayayei* could mostly not afford the costs of biopsy, diagnostic LEEP, or histopathology, on counseling, those who showed major changes on VIA preferred to undergo thermal coagulation in the same visit and setting. Eligibility for thermal coagulation was assessed in strict accordance with the WHO guidelines [38]: no suspicion of invasive disease at colposcopy *and* an entirely visible lesion without extension into the endocervical canal, *or* a type 1 transformation zone lesion, *or* a type 2 transformation zone lesion for which the probe tip can achieve thorough ablation of the squamocolumnar junction (reach the upper limit of the transformation zone). Women who showed minor changes on VIA (*n* = 1) would be counselled about the option of conservative management (rescreening in 6 months to 1 year) because such lesions (probably CIN I) could spontaneously regress or to have immediate treatment with thermal ablation which could have been overtreatment, but potentially lifesaving for those who might never get the opportunity to be screened again in their lifetime. Women who tested negative for hr-HPV and showed no cervical abnormalities on VIA were advised to repeat screening after five years. *Kayayei* who tested hr-HPV positive without corresponding cervical lesions were counseled to repeat HPV DNA testing in 1 year at a nearby facility or the CCPTC. Women who fell into these last two categories were counselled after we ran the HPV DNA tests at our central laboratory, not on site.

**Table 3:** Exploratory logistic regression analyses of sociodemographic and clinical factors associated with hr-HPV positivity among migrant head porters who underwent cervical screening via concurrent hr-HPV DNA testing and VIA

	Univariate analysis				Multivariable analysis		
Variable	OR	95% CI	*p*-value		aOR	95% CI	*p*-value
Age, years (continuous)	0.98	0.87–1.10	0.757		0.80	0.65–1.00	0.050
Age group, years							
<23	1.46	0.50–4.31	0.491		-		
≥23	Ref.	Ref.	Ref.		-		
No. of prior pregnancies (discrete)	1.18	0.80–1.74	0.396		-		
No. of prior pregnancies							
<2	0.42	0.14–1.26	0.123		0.11	0.02–0.69	0.018*
≥2	Ref.	Ref.	Ref.		Ref.	Ref.	Ref.
No. of children (discrete)	1.05	0.36–3.04	0.924		-		
No. of children							
0	Ref.	Ref.	Ref.		-		
≥1	0.91	0.24–3.36	0.883		-		
Marital status							
Married	Ref.	Ref.	Ref.		-		
Single/has a steady partner/ divorced/ widowed	1.09	0.35–3.35	0.882		-		
Education level							
None	Ref.	Ref.	Ref.		-		
Elementary school	0.52	0.13–2.10	0.361		-		
Secondary school	0.65	0.18–2.36	0.510		-		
Monthly income level							
<100	Ref.	Ref.	Ref.		-		
≥100	0.78	0.27–2.31	0.657		-		
Source of funds for medical bill payment							
Self	0.96	0.26–3.62	0.957		-		
NHIS	0.96	0.26–3.62	0.957		-		
Past contraceptive use	1.76	0.60–5.23	0.305		3.58	0.97–13.21	0.056
Abnormal vaginal discharge	1.41	0.36–5.53	0.623		-		

hr-HPV, high-risk human papillomavirus; CI, confidence interval; OR, crude odds ratio; aOR, adjusted odds ratio; NHIS, National Health Insurance Scheme; Ref., reference category; VIA, visual inspection with acetic acid

* Statistically significant

## Discussion

We conducted this pilot study to explore the prevalence of hr-HPV infection and cervical lesions among *kayayei* plying their trade in the Greater Accra Region and to describe our approach to triaging and treating cervical lesions found among them. It is logistically challenging to provide cervical precancer screening and treatment in low-resource settings. Major barriers include women’s time and the cost of transportation to visit a clinic, laboratory procedures that make it nearly impossible to get same-day results, and unavailability of ablative therapy [39]. Another challenge is loss to follow-up, such that any strategy aimed at giving priority to screening, triage, and treatment in a single visit or at the setting would result in a greater reduction in cervical cancer incidence [40]. While *kayayei* in Ghana are recognized as a vulnerable population [14, 15, 18–20, 22, 23, 26, 30] faced with numerous sexual and social inequalities [21, 27, 31], there has been no prior study, to our knowledge, on cervical screening services in this population. Here, cervical precancer screening was performed using HPV DNA testing in line with the WHO’s call for screening via this approach in resource-limited countries like Ghana [41]. In addition, we performed VIA (and EVA colposcopy) in a single session, to reduce the need to contact HPV-positive head porters to come for VIA, and reduce loss to follow-up.

Although only 63 women were screened in this cohort, the overall hr-HPV positivity rate among the *kayayei* (33.3%; 95% CI, 21.7–46.7) was similar to those reported among community-dwelling women in the North Tongu District of Ghana [42] (32.3%; 95% CI, 30.2–34.5) and women attending Cervicare or reproductive health clinics in Ghana [43] (35.0%; 95% CI, 29.6–40.4). It should, however, be borne in mind that certain methodological differences make it challenging to compare the prevalence identified among the migrant head porters to these previously studied cohorts. In addition to the recognized high-risk HPV genotypes (HPV 16, 18, 31, 33, 35, 39, 45, 51, 52, 56, 58, and 59), the qualitative version of the MA-6000 test we used also categorizes probable high-risk HPV genotypes (HPV 66 and 68) and one potential high-risk HPV type (HPV 53) under *other* hr-HPV types. Due to financial limitations, we were unable to perform full genotyping tests for *kayayei* who screened HPV positive for any type. Interestingly, however, the hr-HPV positivity rate was nearly double that reported among community-dwelling women in Ibadan, Nigeria [44] (19.7%; 95% CI, 17.2–22.4) when HPV 53, 66, and 68 were categorized under ‘hr-HPV’. The hr-HPV detection rate also exceeded values of 10.7% reported among another group of women attending an outpatient gynecology clinic in Accra [45] and 13.9% (95% CI, 9.2–20.4) reported among pregnant Ghanaian women in the Western region [46]. Thus, notwithstanding, given that the head porters were much younger than the previously studied Ghanaian cohorts, we expect the age-standardized positivity rates for recognized hr-HPV genotypes to be much higher.

Ghana has no national cervical cancer screening register or program or national HPV vaccination program. To further compound the high burden of hr-HPV infection found among the head porters, prior research has shown that *kayayei* have limited access to healthcare and tend to seek care in avenues outside the general healthcare system, including drug peddlers, herbalists, and chemist shops [21]. This is understandable, as with limited savings, *kayayei* do not have the financial muscle to pay for healthcare outside the support provided by the NHIS and non-governmental organizations [21]. Even though close to 80% of our study cohort had NHIS coverage compared to the 45% reported by Shamsu–Deen and Adadow [21], cervical screening services are costly and are not currently covered by the NHIS in Ghana [47]. In addition to the prevalence estimates highlighted in this study, a favorable cervical screening policy should aim at preventing inequalities in the follow-up of pathological results, which could be challenging for this group of women who are out of touch with the health system. Again, a low rate of contraceptive (including condom) use among *kayayei* has been reported, with key barriers being financial implications from the meagerness of their earnings, as well as disapproval from male partners [27]. Given that a majority of the *kayayei* earned below GH¢ 250 (32.1 USD) compared to the national minimum wage of GH¢ 354.6 around the time we conducted this screening outreach [48] (45.5 USD) (based on a median conversion rate of 1 USD = 7.8 in May 2022), it is not surprising that screen positives opted for immediate management with thermal ablation under a ‘mobile colposcopy and treat’ approach. Trapped in this affordability dilemma, the *kayayei* were likely forced to prioritize daily or ‘survival’ needs, making it logical to push for cervical screening costs to be rolled into the NHIS, particularly to cover such vulnerable groups. This recommendation is also not out of order since prior studies have reported high costs as a hindrance to cervical screening uptake [47].

### Limitations and strengths

Our study had some limitations. First, due to its pilot nature, the small sample size restricted our ability to present more granular details pertaining to risk factors for hr-HPV positivity in the study cohort. In addition, the limited sample size restricted the generalizability of the findings to the population of over 100,000 *kayayei* in Ghana. In hindsight, we may have been able to extend our cohort size through proper community entry via the *Kayayei* Youth Association, rather than through self-introduction and contacting volunteers directly at the marketplace. Further, the HIV prevalence presented here was likely under-reported as the data were collected via self-report and not based on field testing. Because this project had limited funding, the women who showed major lesions on VIA who would under normal circumstances have paid to have cervical biopsies and histopathology rather had upfront thermal coagulation. Therefore, we were unable to determine the histopathological diagnoses for the cervical lesions seen which were treated by ablation in this context. Finally, we did not perform this work in a research context. The *kayayei* were provided with the same level of care available to women seeking care at the CCPTC, Battor. Thus, having used the same prescreening forms for general attendees, we could not obtain data specific to *kayayei* to assess risk factors such as duration as a head porter, reasons for migrating to the south, dangers faced in their line of work (e.g., prior rape, place of residence, and risks faced in search of decent accommodation), as well as coping strategies for navigating the health system.

Despite these limitations, the findings of the present pilot study add to the body of evidence and discourse pertaining to cervical precancer screening in marginalized and vulnerable populations in Ghana. *Kayayei* represent a distinctive group in terms of sexual health needs and access to the healthcare system for general and cervical screening purposes. Our findings are expected to serve as a basis for further more rigorous investigations into how the adverse environmental challenges faced by migrant head porters affect their exposure to hr-HPV types, and thus their ability to obtain cervical screening services and follow-up care.

## Conclusions

In this paper, we present, for the first time, pilot estimates of hr-HPV prevalence and cervical lesions among female migrant head porters (*kayayei*) in Ghana. In this relatively young cohort with a high hr-HPV infection rate of 33.3% and 8.3% of women showing cervical lesions on visual inspection, we posit that *kayayei* may have an increased risk of developing cervical cancer if they continue to have difficulty accessing cervical screening services. Thus, national cervical screening guidelines should include migrant head porters and other vulnerable groups to reduce the incidence of cervical cancer. Given the pilot nature of the present study with a limited sample size, further studies with larger samples more representative of this vulnerable group of migrant head porters are warranted and may clarify our results and aid in planning appropriate preventive strategies tailored to them.

## Data Availability

The datasets used and/or analyzed during the current study are available from the corresponding author on request.

## Abbreviations

CCPTC: Cervical Cancer Prevention and Training Centre
CI: confidence interval
EVA: Enhanced Visual Assessment
hr-HPV: high-risk human papillomavirus
LEEP: loop electrosurgical excision procedure
NHIS: National Health Insurance Scheme
VIA: visual inspection with acetic acid
WHO: World Health Organization
